# Appropriate determination of the surgical transepicondylar axis can be achieved following distal femur resection in navigation-assisted total knee arthroplasty

**DOI:** 10.1186/s43019-021-00123-1

**Published:** 2021-11-10

**Authors:** Sang Jun Song, Hyun Woo Lee, Kang Il Kim, Cheol Hee Park

**Affiliations:** Department of Orthopaedic Surgery, College of Medicine, Kyung Hee University, 23 Kyunghee-daero, Dongdaemun-gu, Seoul, 130-872 South Korea

## Abstract

**Background:**

Many surgeons have determined the surgical transepicondylar axis (sTEA) after distal femur resection in total knee arthroplasty (TKA). However, in most navigation systems, the registration of the sTEA precedes the distal femur resection. This sequential difference can influence the accuracy of intraoperative determination for sTEA when considering the proximal location of the anatomical references for sTEA and the arthritic environment. We compared the accuracy and precision in determinations of the sTEA between before and after distal femur resection during navigation-assisted TKA.

**Methods:**

Ninety TKAs with Attune posterior-stabilized prostheses were performed under imageless navigation. The sTEA was registered before distal femur resection, then reassessed and adjusted after distal resection. The femoral component was implanted finally according to the sTEA determined after distal femur resection. Computed tomography (CT) was performed postoperatively to analyze the true sTEA (the line connecting the tip of the lateral femoral epicondyle to the lowest point of the medial femoral epicondylar sulcus on axial CT images) and femoral component rotation (FCR) axis. The FCR angle after distal femur resection (FCRA-aR) was defined as the angle between the FCR axis and true sTEA on CT images. The FCR angle before distal resection (FCRA-bR) could be presumed to be the value of FCRA-aR minus the difference between the intraoperatively determined sTEAs before and after distal resection as indicated by the navigation system. It was considered that the FCRA-bR or FCRA-aR represented the differences between the sTEA determined before or after distal femur resection and the true sTEA, respectively.

**Results:**

The FCRA-bR was −1.3 ± 2.4° and FCRA-aR was 0.3 ± 1.7° (*p* < 0.001). The range of FCRA-bR was from −6.6° to 4.1° and that of FCRA-aR was from −2.7° to 3.3°. The proportion of appropriate FCRA (≤ ±3°) was significantly higher after distal femur resection than that before resection (91.1% versus 70%; *p* < 0.001).

**Conclusions:**

The FCR was more appropriate when the sTEA was determined after distal femur resection than before resection in navigation-assisted TKA. The reassessment and adjusted registration of sTEA after distal femur resection could improve the rotational alignment of the femoral component in navigation-assisted TKA.

**Level of evidence:**

IV.

## Introduction

Appropriate rotation of the femoral component is critical for a successful outcome after total knee arthroplasty (TKA) [1, 2]. The malrotation of the femoral component is known to be associated with postoperative complications including patellofemoral maltracking, anterior knee pain, stiffness, flexion instability, post-cam impingement, polyethylene wear and subsequent osteolysis, and component loosening [3]. Various references have been suggested to help surgeons discern the proper rotation of the femoral component to pursue intraoperatively, and the surgical transepicondylar axis (sTEA) has been demonstrated as the most reliable reference for femoral component rotation (FCR), despite difficulty with its identification [2].

Navigation is recognized as a useful tool by which to reproducibly position components with the desirable coronal and sagittal alignment in the TKA procedure [4, 5]. However, it remains under debate whether the rotational alignment can be further improved [6, 7]. The accuracy of navigation-assisted TKA depends upon the appropriate registration of bony landmarks, and it is known that registration errors for sTEA can occur frequently, especially in procedures guided by imageless navigation because of difficulty with identification [4].

Many surgeons have determined the sTEA after distal femur resection [8]. However, in most navigation systems, the registration of the sTEA precedes the distal femur resection [9, 10]. This sequential difference can influence the accuracy of intraoperative determination for sTEA when considering the proximal location of the anatomical references for sTEA and the arthritic environment [6]. To our knowledge, no previous study has addressed this issue.

The purpose of the present study was to compare the accuracy and precision between intraoperative determinations of sTEA before and after distal femur resection during navigation-assisted TKA. It was hypothesized that the determination of sTEA after distal femur resection would be more appropriate than that before resection.

## Materials and methods

### Patients

The present study was conducted prospectively, and data were reviewed retrospectively. Ninety TKAs using Attune posterior-stabilized prostheses (Depuy Synthes, Warsaw, IN, USA) were performed under the guidance of an imageless navigation system (Knee 3, BrainLAB, Heimstetten, Germany) between July 2019 and September 2019. All TKAs were performed by a senior surgeon with surgical experience of more than 2000 cases of conventional TKA and more than 300 cases of navigation-assisted TKA.

The inclusion criterion was primary TKA due to Kellgren–Lawrence grade 4 degenerative osteoarthritis with varus deformities. The exclusion criteria were inflammatory arthritis; a history of knee infection, fracture, dislocation, ligament injury, reconstructive ligament surgery, or high-tibial osteotomy; and knee with extra-articular deformity. A knee with a valgus deformity was also excluded due to the possible deterioration of anatomy of the distal femur [11].

The preoperative demographics are presented in Table 1. This study was approved by the institutional review board. Informed consent was obtained from all patients before commencing the review.

**Table 1:** Preoperative demographic data

	Number of cases or mean ± standard deviation
Number of cases	90
Age (years)	73.4 ± 5.6
Sex (female/male)	88/2
Body mass index (kg/m^2^)	26.4 ± 3.3
Side (right/left)	48/42
Preoperative range of motion (°)	122.6 ± 20.3
Follow-up period (months)	12.1 ± 2.7

### Surgical techniques, including intraoperative determination of sTEAs, and rehabilitation

All TKA procedures were performed with a modified measured resection technique under navigation guidance. A tourniquet was applied during the procedure. The medial parapatellar approach was adopted with a midline skin incision, and the patella was everted. The reference arrays were placed on the medial side of the distal femur and proximal tibia. The hip center was registered kinematically with hip circumduction. Other anatomical landmarks were registered with point referencing before bone resections.

**Fig. 1:**
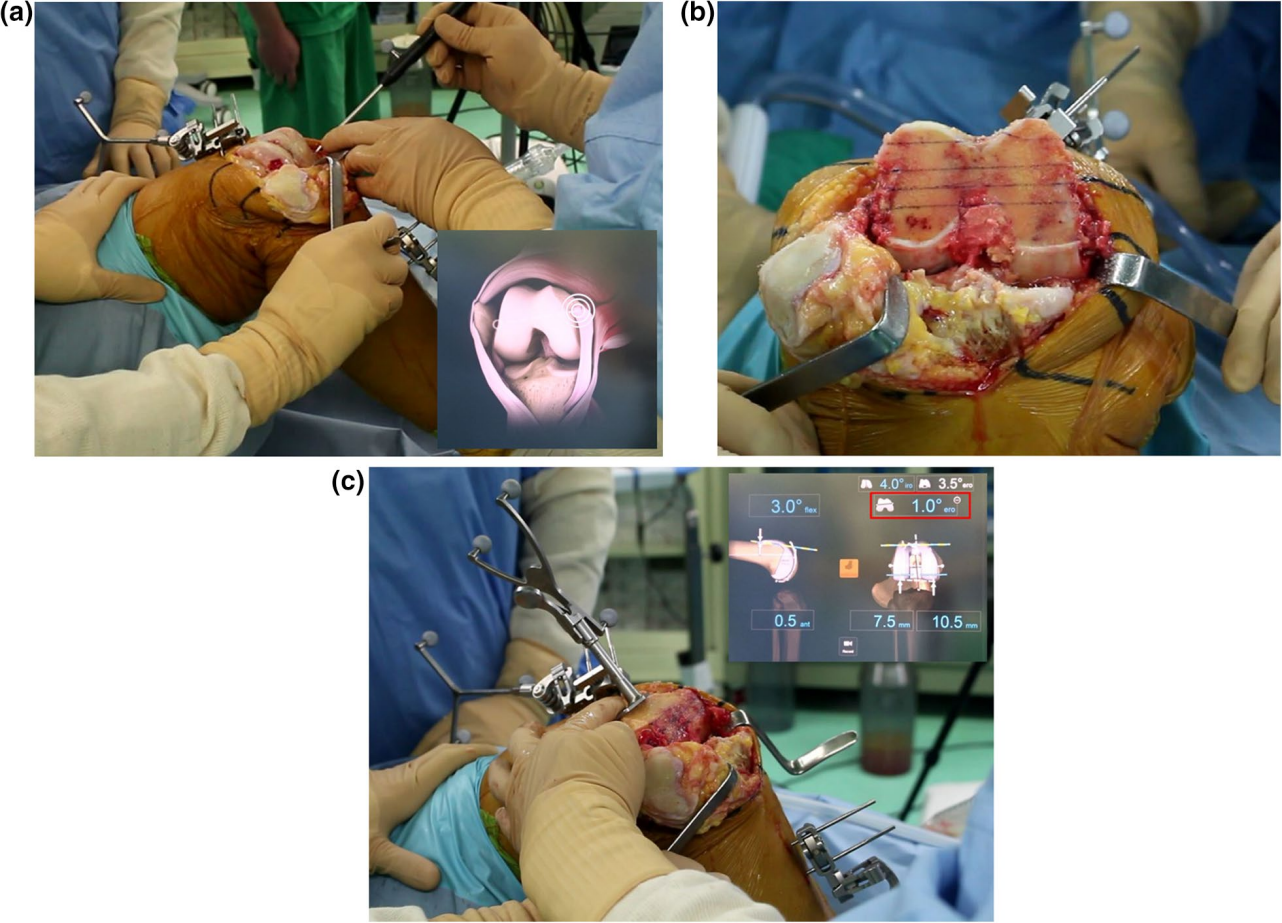
Intraoperative determination of the surgical transepicondylar axis (sTEA) before and after distal femur resection. **A** The registration of sTEA as determined before distal femur resection. **B** The determination of sTEA after distal femur resection. The lowest line is the intraoperatively determined sTEA connecting the tip of the lateral femoral epicondyle to the lowest point of the medial femoral epicondylar sulcus after distal femur resection. The remaining lines are ancillary lines drawn for the accurate rotational alignment of the anteroposterior femur cutting guide. **C** The verification process for anteroposterior femur resection. The navigation indicates the difference between intraoperatively determined sTEAs before and after distal femur resection (red square)

To register sTEA as a rotational axis of the femoral component, we identified the medial epicondylar sulcus and most prominent point of the lateral epicondyle (Fig. 1A). Following this registration process, distal femur resection was performed and, subsequently, the sTEA was reassessed and adjusted (Fig. 1B). The rotational alignment of the anteroposterior (AP) femoral cutting guide was finally decided according to the sTEA determined after distal femur resection and AP resection of the distal femur was performed. During the verification process after AP femoral resection, the navigation system indicated the difference between the final rotational alignments of the femoral component and the sTEA registered prior to distal femur resection; this value indicated the difference between intraoperatively determined sTEAs before and after distal femur resection (Fig. 1C, red square). External rotation of sTEA determined after distal resection relative to that before resection was denoted as a positive value, and internal rotation was denoted as a negative value.

Tibial resection was performed to establish a posterior slope of 2° under navigation guidance. Soft-tissue balancing could be evaluated continuously at every degree within the range of motion (ROM) using the Knee 3 navigation software. If the mediolateral gap differed by more than 2 mm after bone resection, the tibial cut surface or ligament balance was adjusted. All patellae were resurfaced. Patellofemoral articulation was carefully evaluated with the no-thumb technique. All components were implanted on cleaned and dried cut surfaces using a full cementation technique.

### Radiographic evaluation

**Fig. 2:**
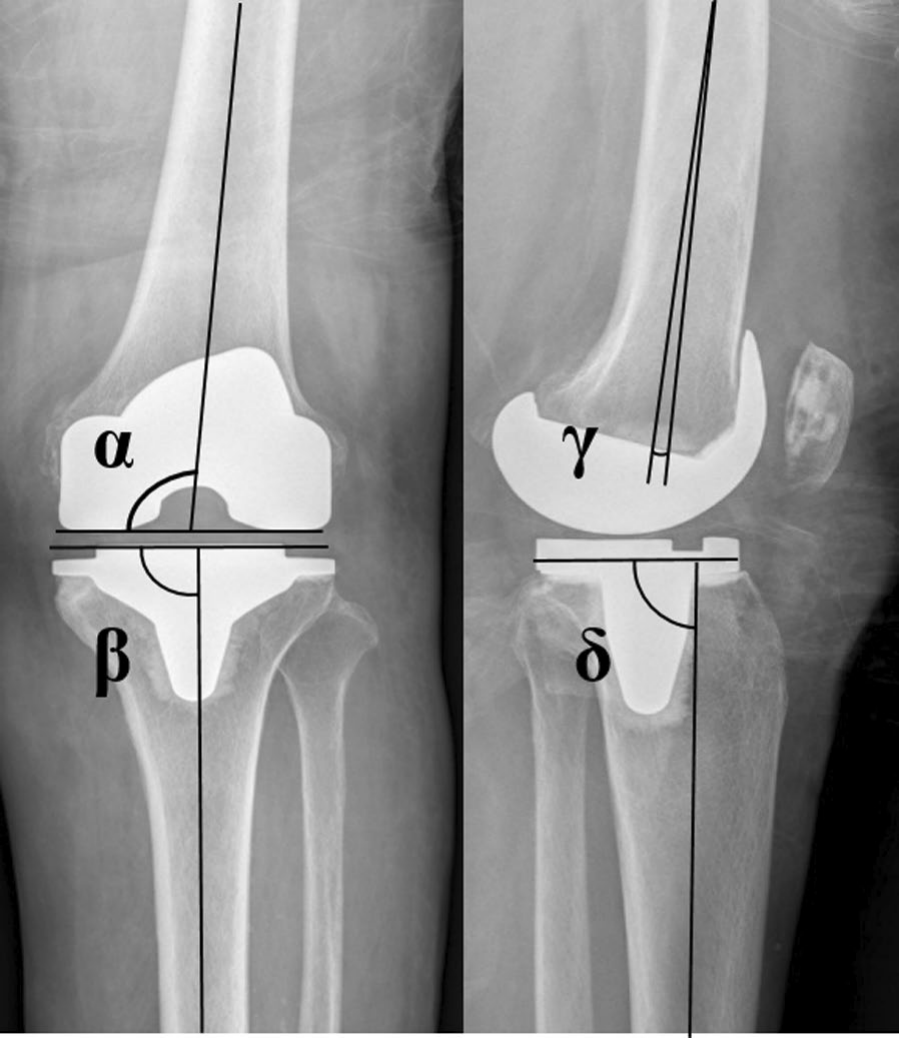
The positions of components evaluated with the Knee Society radiological evaluation method

Pre- and postoperative AP and lateral radiographs and orthoroentgenograms (i.e., full-length standing AP radiographs) were obtained to assess limb alignment and component positioning. The pre- and postoperative mechanical axes were defined each as the angle between the femoral and tibial mechanical axes on orthoroentgenograms. Detailed analyses of AP and lateral radiographs were performed to evaluate the positions of components with *α*, *β*, *γ*, and *δ* angles using the Knee Society radiological evaluation method [11] (Fig. 2). The radiographic parameters were measured preoperatively and at 1 week after surgery.

**Fig. 3:**
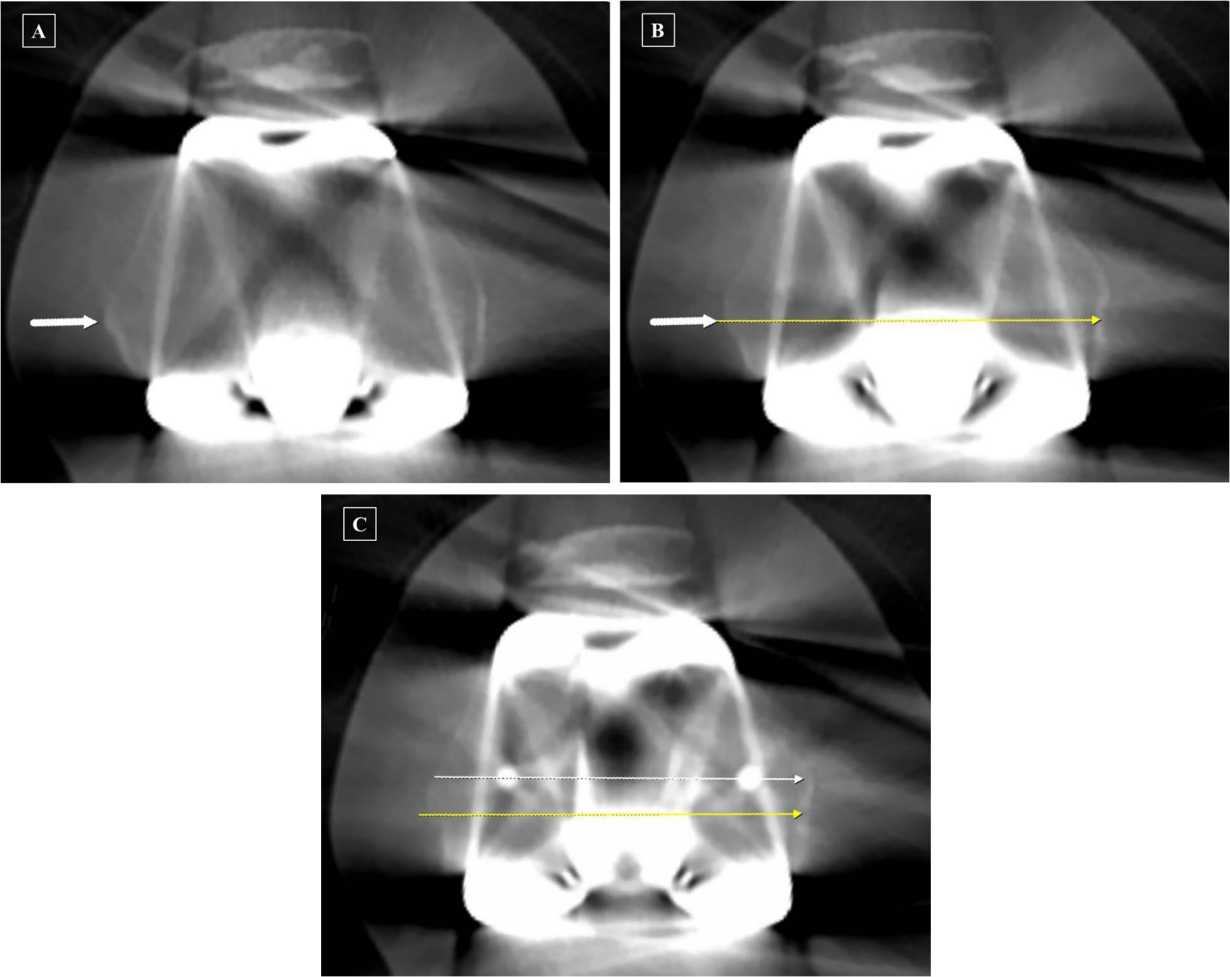
Radiographic determination of surgical transepicondylar axis (sTEA) and femoral-component rotational axis on the axial view of computer tomography. **A** White arrow: the tip of the lateral epicondyle. **B** Pasted white arrow: described above; the yellow line indicates the sTEA. **C** Pasted yellow line: described above; white line: the femoral-component rotational axis

Computer tomography (CT) was performed at 4 weeks postoperatively to analyze the true sTEA and FCR axis. A 64 multislice CT scanner (General Electric, Boston, MA, USA) with a collimation value of 0.75 was used. CT imaging was performed perpendicular to the long axis of the femur with a slice thickness of 0.63 mm [6]. The true sTEA was defined as the line connecting the tip of the lateral femoral epicondyle and the lowest point of the medial femoral epicondylar sulcus on axial CT images. Given the possibility that the above two reference points were not visible in the same axial image [6], the following evaluation technique was used: an arrow indicating the tip of the lateral femoral epicondyle was made on the image where the tip was clearly visible, and this arrow was copied (Fig. 3A); this copied arrow was then pasted at the same spot on the image where the lowest point of the medial femoral epicondylar sulcus was clearly visible, and the line connecting the lowest point of the sulcus and arrow was defined as the true sTEA (Fig. 3B, yellow line). The FCR axis was defined as the line connecting the two peg centers of the femoral component (Fig. 3C, white line).

The quality of radiographs and CT scans were able to be improved by the protocol of standardizing the positioning of the knee. The true AP radiographs and orthoroentgenograms were taken with the patient standing with their knee fully extended and their feet slightly internally rotated to ensure forward placement of knees [12]. For the true lateral radiographs, an effort was made to achieve superimposition of the medial and lateral femoral condyles of the distal femur on the radiographs [13]. When performing CT imaging, the patient was positioned supine with full extension and forward placement of the knee.

The collected images were transferred digitally to a picture archiving and communication system (PACS) (Infinitt, Seoul, Korea). Assessments were conducted on a 61-cm (24-inch) monitor (SyncMaster 2494HMN; Samsung, Seoul, South Korea) in portrait mode using the PACS software, which boasted “copy” and “paste” functions. The minimum difference in an angle that the PACS software could detect was 0.1° [14].

To minimize observation bias, two orthopedic surgeons who did not participate in the surgeries repeatedly performed all radiographic measurements at an interval of 2 weeks. The intra- and interobserver reliabilities of all measurements were assessed using the intraclass correlation coefficient, and all values were found to be greater than 0.8. Thus, the average values between the two investigators were used for final analysis.

### Evaluation of FCR

FCRA after distal femur resection (FCRA-aR) was defined as the angle between the FCR axis and the true sTEA as evaluated on the CT images (Fig. 3C, angle between the two lines). External rotation of the FCR axis relative to the true sTEA was denoted as a positive value, while internal rotation was denoted as a negative value. The FCRA-aR refers to the difference between the sTEA adjusted after distal femur resection and the true sTEA.

The FCRA before distal femur resection (FCRA-bR) could be presumed to be the value of FCRA-aR minus the intraoperative difference between the sTEAs determined before and after distal femur resection (Fig. 1C, red square), which was stored in the navigation system during the procedure (FCRA-bR = FCRA-aR − intraoperative difference between sTEAs determined before and after distal femur resection). The FCRA-bR is a value indicating how much a femoral component would rotate relative to the true sTEA if the femoral component was implanted according to the registered sTEA before distal femur resection. Accordingly, the FCRA-bR refers to the difference between the sTEA determined before distal femur resection and the true sTEA.

### Statistical analysis

The pre- and postoperative radiographic results were compared using a paired *t*-test. The degree of accuracy regarding the intraoperative determination of sTEA before and after distal femur resection was evaluated with an average value of FCRA; the average value of FCRA-bR and FCRA-aR was compared using the paired *t*-test. The precision of intraoperative sTEA determination was evaluated with the variability of FCRA as presented, with standard deviation (SD) and range values. The proportion of appropriate FCRA (≤ ±3°) before and after distal femur resection was compared using the McNemar test [15]. Statistical analyses were performed using the Statistical Package for the Social Sciences version 18.0 (IBM Corporation, Armonk, NY, USA), and *p*-value of less than 0.05 was considered to be statistically significant.

A priori power analysis was performed based on preliminary data of an initial group of 30 cases to determine the minimum sample size affording sufficient power, with FCRA considered as the primary outcome. The analysis was performed to achieve power for detecting significant differences between the FCRA-bR and FCRA-aR. The mean ± SD values of FCRA-bR and FCRA-aR were 0.6 ± 2.0 and −1.1 ± 2.3, respectively, and the correlation between FCRA-bR and FCRA-aR was 0.782 (*p* < 0.001) in the preliminary group. The alpha and power values were set at 0.05 and 80%, respectively. The results of sample-size calculation showed the need for at least eight cases. Consequently, our sample size was determined to have sufficient power.

## Results

Radiographically, the MA was corrected from varus 12.4° to varus 1.3° (*p* < 0.001), and the overall positions of all components were appropriate (Table 2).

**Table 2:** Pre- and postoperative radiographic results

	Preoperative	Postoperative
Mechanical axis (°)*	Varus 12.3 ± 4.1	Varus 1.3 ± 2.0
Position of components (°)		
*α* angle		96.1 ± 1.6
*β* angle		90.0 ± 1.1
*γ* angle		2.5 ± 2.0
*δ* angle		88.3 ± 1.8

Data are presented as mean ± standard deviation; *p-value between preoperative and postoperative mechanical axes was < 0.001

**Fig. 4:**
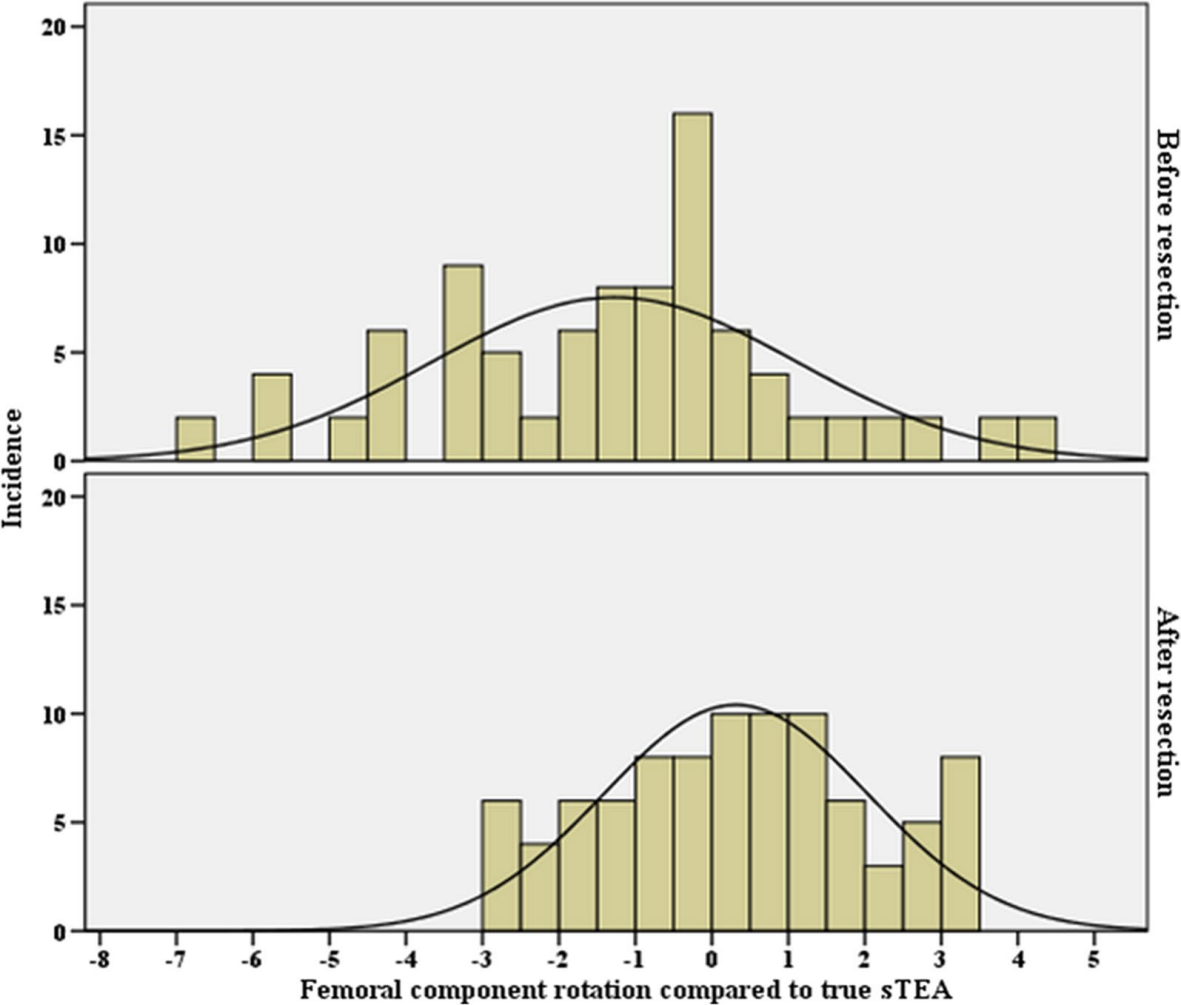
The distribution of femoral component rotation angle relative to the true surgical transepicondylar axis (sTEA) before and after distal femur resection

The average FCRA-bR was −1.3°, and the average FCRA-aR was 0.3° (*p* < 0.001). The SD was 2.4° in FCRA-bR and 1.7° in FCRA-aR. The range of FCRA-bR was from −6.6° to 4.1°, and that of FCRA-aR was from −2.7° to 3.3° (Fig. 4). The proportion of appropriate FCRA outcomes was significantly higher after distal femur resection relative to before resection (91.1% versus 70%; *p* < 0.001) (Fig. 4).

## Discussion

The most important finding of the present study was that accuracy and precision in the intraoperative determination of sTEA were better after distal femur resection than prior to resection in navigation-assisted TKA. The proportion of appropriate FCRA within ± 3° was also significantly higher when the rotation was aligned with the sTEA determined after distal femur resection.

The sTEA is the axis connecting the lateral femoral epicondylar prominence and the central sulcus of the medial femoral epicondyle and is widely accepted as the gold standard for FCR [2, 3]. Previous studies have suggested sTEA to be the landmark that best coincides with the functional axis of the knee during 0° to 90° flexion [2, 3]. Additionally, it has been reported that the flexion space is more reliably balanced when adopting the sTEA for FCR [16].

However, the reproducible and accurate identification of the sTEA intraoperatively can be difficult to complete in severely osteoarthritic knees because it relies on the palpation of deformed and broad osseous landmarks covered with thick soft tissues and massive osteophytes in arthritic knees [2]. Jeroch et al. [17] investigated the interobserver reproducibility of sTEA, comparing the location of the medial epicondylar sulcus and lateral epicondylar prominence marked by different surgeons, and demonstrated that the range of positioning identified by surgeons varied by 22.3 mm for the medial sulcus and 13.8 mm for the lateral epicondyle. Kinzel et al. [18] reported that 75% of femoral components were aligned within 3° of the sTEA on CT images, with a wide range of error from 6° of external rotation to 11° of internal rotation of the femoral component.

Navigation-assisted TKA has demonstrated the capacity to improve TKA alignment in the coronal and sagittal planes [6]. However, it is unclear whether the rotational alignment of the TKA components can be improved, especially when using an imageless navigation system [6]. Matziolis et al. [7] reported that the rotational alignment of the femoral component was not improved by navigation itself when determining the FCR according to the intraoperatively registered sTEA. This outcome might have been caused by the fact that the accuracy of sTEA registration fully relied on the correct identification of bony landmarks by the operating surgeon. The accuracy and precision of navigation can be jeopardized by registration error, which can be most evident in the sTEA registration due to the difficulty of intraoperative identification [4]. It cannot be overemphasized that the sTEA needs to be accurately recognized and registered in navigation-assisted TKA to ensure optimal outcomes.

Generally, the registration of sTEA is performed before distal femur resection in the navigation system, which is different from the process during conventional TKA procedures, which involve determining the sTEA after distal resection [8–10]. Based on the following reasons, we hypothesized that it would be more advantageous to determine sTEA intraoperatively after distal femur resection. First, anatomical landmarks for sTEA are located fairly proximal from the joint surface. In particular, the medial epicondylar sulcus is known to be located more proximal even compared with the medial epicondyle [6]. Distal femur resection can facilitate easier access to the landmarks for sTEA. Second, the landmarks for sTEA can be hidden by hypertrophied synovium and osteophytes in the arthritic knee [6]. After distal resection, both bony landmarks and these obstacles can be better distinguished; the osteophyte or synovium can be removed more during the distal resection, and the cortical margin of landmarks can be clearly seen on the cut bony surface after resection [16]. Lastly, the conduct of patella eversion or lateralization is easy after distal femur resection and allows for sufficient visualization of the lateral femoral epicondyle. The results of the present study indicate that determining the sTEA after distal femur resection was beneficial to ensuring the accurate and precise rotational alignment of the femoral component in navigation-assisted TKA.

Several methods, including the use of previously reported CT information or tibial-first procedures, have been suggested for improving the rotational alignment of the femoral component in navigation-assisted TKA [6, 10]. In addition, we propose an adjustment of the registration for sTEA after distal femur resection. Our suggestion will help to attain a more appropriate degree of FCR in various types of navigation systems. Especially, it is thought that this method will reduce the registration error for a low-volume surgeon without sufficient surgical experience [19].

The present study had several limitations. First, consistent evaluation of FCRA-aR and FCRA-bR in one system (either navigation or CT) would have been a better approach; however, this was difficult to achieve in a clinical situation. With our navigation system, the peg hole could not be registered for FCRA-aR. The landmark intraoperatively determined by the operator before distal femur resection could not be confirmed on postoperative CT. We considered our evaluation method to be the best way by far to conduct this study. Second, FCRA-bR was evaluated under the assumption that the femoral component would be inserted precisely according to the sTEA determined by the surgeon before distal femur resection. However, this assumption could have a limitation: the positioning of the femoral and tibial components, which mainly involves cementation and impaction, can introduce a considerable error in alignment during final implantation, regardless of how accurately the resection planes are made [20]. Thus, a more sophisticated study considering this limitation is required. Third, the measurement of true sTEA following CT imaging was performed on two-dimensional axial CT slices; yet, the sTEA is a three-dimensional structure, and the medial epicondylar sulcus rarely lies in the same axial plane as the tip of the lateral epicondyle [6]. De Valk et al. [1] found that three-dimensional CT ensured a more accurate determination of FCR. To address this concern, we adopted a measurement technique that involves several consecutive axial CT slices. The reliability of our method was confirmed by the realization of good intraclass correlation coefficient values as measured by two investigators. Fourth, there was no control group that determined the final FCR according to the registered sTEA before distal femoral resection. A detection bias reflecting the researcher’s intention can occur in our method for determining sTEA sequentially at the same knee intraoperatively. However, the comparison of the intraoperative determination of sTEAs in identical knees can reveal a side that allowed for more accurate evaluation of the effect of distal femur resection, because it is performed under exactly the same conditions, except for distal femur resection. Finally, the number of measurements performed on CT scans may affect the level of accuracy and precision of the values. Averaging data from two measurements may help to attain mean values closer to the truth, but the SD and range values will tend to decrease.

## Conclusion

The FCR was more appropriate when the sTEA was determined intraoperatively after distal femur resection than before resection in navigation-assisted TKA. The reassessment and adjusted registration of sTEA after distal femur resection could improve the rotational alignment of femoral components in navigation-assisted TKA.

## Data Availability

The datasets generated and/or analyzed during the current study are not publicly available, but they are available from the corresponding author on reasonable request.
